# Gadolinium Retention and Clearance in the Diabetic Brain after Administrations of Gadodiamide, Gadopentetate Dimeglumine, and Gadoterate Meglumine in a Rat Model

**DOI:** 10.1155/2019/3901907

**Published:** 2019-05-05

**Authors:** Shu-ting Wang, Zheng-xu Hua, Dong-xiao Fan, Xin Zhang, Ke Ren

**Affiliations:** ^1^Department of Radiology, The First Affiliated Hospital, China Medical University, Shenyang, Liaoning 110001, China; ^2^Key Laboratory of Imaging Diagnosis and Interventional Radiology of Liaoning Province, Shenyang, Liaoning 110001, China; ^3^Department of Orthopedic Surgery, The First Affiliated Hospital, China Medical University, Shenyang, Liaoning 110001, China

## Abstract

**Purpose:**

To evaluate gadolinium (Gd) retention and clearance in the brain of diabetic rats after administrations of gadodiamide, gadopentetate dimeglumine, and gadoterate meglumine.

**Materials and Methods:**

Both diabetic rats (n = 52) and normal rats (n = 52) intravenously received 20 injections of 0.6 mmol Gd/kg gadodiamide, gadopentetate dimeglumine, gadoterate meglumine, or saline. Both diabetic rats and normal rats were divided into 2 subgroups of 24 and 28 rats for the 7-day and 42-day evaluations (i.e., they were sacrificed at 7 days (n = 6 per group) and 42 days (n = 7 per group)), respectively, after the last injection. For the 7-day subgroup, 6 rats were euthanized for inductively coupled plasma mass spectrometry (ICP-MS) analysis. For the 42-day subgroup, 6 rats underwent T1-weighted magnetic resonance imaging (MRI) and ICP-MS, and 1 rat was analyzed by transmission electron microscopy (TEM).

**Results:**

The T1 enhancements in the deep cerebellar nuclei (DCNs) of diabetic rats were lower than those of normal rats in both linear Gd-based contrast agent (GBCA) groups (p < 0.05). The average Gd concentrations in the brains of diabetic rats were significantly lower than those of healthy rats in both the short-term groups and long-term groups (*p < *0.05). The highest Gd retentions were in the olfactory bulb, DCN, and striatum with gadodiamide. Compared with the results obtained 7 days after the last injection, the residual Gd concentrations of the 42-day subgroups in the brains of diabetic rats showed no significant difference in both linear GBCA groups (*p>*0.05).

**Conclusions:**

Compared with normal rats, the diabetic status decreased the residual Gd concentrations in the brain after multiple administrations of gadodiamide, gadopentetate dimeglumine, and gadoterate meglumine. The clearable fraction of Gd in the brain was eliminated faster in diabetic rats than in normal rats.

## 1. Introduction

Gadolinium (Gd)-based contrast agents (GBCAs) have been widely used in enhanced magnetic resonance imaging (MRI) examinations since the 1980s. They have been considered relatively safe due to the low incidence of serious adverse effects until several reports in 2006 associated nephrogenic systemic fibrosis (NSF) with multiple GBCA exposure [1, 2]. Recent human and animal model studies have revealed increased signal intensities in the deep cerebellar nucleus (DCN) and globus pallidus on unenhanced T1-weighted MR images following repeated intravenous administrations of GBCAs. Further studies demonstrate that detectable Gd deposits in brain tissues are associated with multiple administrations of GBCAs, especially linear GBCAs with lower intrinsic stability [3–5]. McDonald et al., Smith et al. and Lohrke et al. showed that no histopathological findings were found in the brains of patients and healthy rats with Gd retention [6–8]. However, the potential risks of these deposits remain unclear, and the Food and Drug Administration (FDA) and the European Medicines Agency have attached great importance to GBCA-induced Gd retention [9, 10].

The mechanism of GBCA accumulation in the brain remains unclear. One potential pathway of GBCA permeation into the brain is through the blood-brain barrier (BBB). For patients with diseases resulting in local breakdown of the BBB, such as brain tumors and inflammation, GBCAs can pass the impaired BBB and accumulate in brain tissues [3, 6]. Another possible route is infiltration from the blood into the cerebrospinal fluid (CSF). The CSF Gd concentration was high 4.5 h after GBCA injection, as determined by inductively coupled plasma mass spectrometry (ICP-MS) [11], indicating that GBCAs might have passed through the blood-CSF barrier. It was postulated that once it permeated into the CSF space, the GBCA would enter the glymphatic system [12].

Diabetes mellitus (DM) is one of the most challenging endocrine disorders across the world and is often accompanied by other disorders, such as tumors, chronic liver diseases, and vascular diseases, requiring repeated enhanced MRI examinations. DM is associated with numerous complications, including diabetic nephropathy and cognitive deficits. A recent study showed that animals with moderate chronic renal disease had higher Gd concentrations in tested brain tissues than healthy animals [13]. Diabetic nephropathy is one of the major causes of renal failure, which may delay GBCA clearance. Furthermore, DM has been shown to progressively disrupt BBB integrity and function [14]. Diabetes is also associated with impairment of the glymphatic system, which has recently been shown to eliminate potential neurotoxic waste products, including GBCAs from the interstitial space [15]. Therefore, it is of particular significance to investigate Gd retention and clearance in diabetic brains compared with healthy brains. To date, no study has assessed whether and how Gd is deposited in the diabetic status.

The purpose of this study was therefore to evaluate the Gd retention and clearance in the brain of diabetic rats after administrations of gadodiamide, gadopentetate dimeglumine, and gadoterate meglumine.

## 2. Materials and Methods

### 2.1. Animal Model

All the animal experiments were approved by the Animal Ethics Committee of China Medical University and were carried out in accordance with the local Guidelines for the Care and Use of Laboratory Animals. Male Wistar rats (220-240 g) were obtained from the Animal Department of China Medical University. The animals were housed in standard laboratory conditions with free access to water and food under a 12:12 h light-dark cycle. The rats were kept 7 days for acclimatization before the formal experiment.

Animals were fasted for 16 h, and the DM model was induced by the method of Motawi, T. K. et al. [16]. Briefly, streptozotocin (STZ) was dissolved in 0.1 M citrate buffer (pH = 4.4) and was injected intraperitoneally at a single dose of 60 mg/kg body weight. After 3 days of STZ injection, the rats were fasted for 8 h, and the blood glucose level was measured. Rats with a blood glucose level >16.7 mmol/L were considered to be diabetic.

### 2.2. Experimental Design

**Figure 1:**
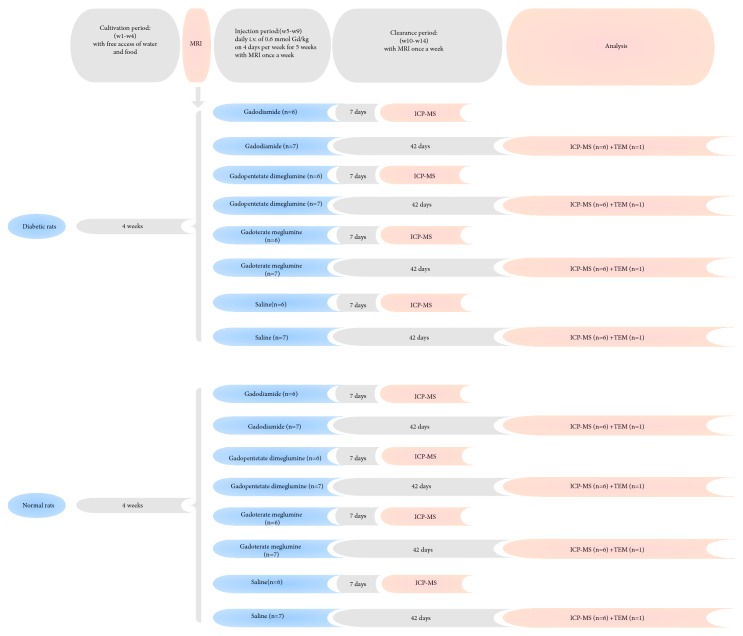
Study schemes. Both diabetic rats and healthy rats received 20 daily administrations of GBCAs and were allocated to 2 subgroups based on the time-point for subsequent analysis. For the 7-day subgroup, 6 rats underwent ICP-MS. For the 42-day subgroup, 6 rats underwent T1-weighted MRI (once a week) and ICP-MS, and 1 rat was analyzed by TEM.

Both diabetic rats (n = 52) and normal rats (n = 52) were maintained routinely for 4 weeks. Afterwards, both groups of rats were randomly allocated to gadodiamide (linear and nonionic GBCA, Omniscan, 500 mmol Gd/L; GE Healthcare), gadopentetate dimeglumine (linear and ionic GBCA, Magnevist, 500 mmol Gd/L; Bayer Healthcare), gadoterate meglumine (macrocyclic and ionic GBCA, Dotarem, 500 mmol Gd/L; Guerbet), and control (0.9% saline solution) groups (n = 13 per group). The rats received 4 daily tail intravenous injections of GBCA or saline per week for 5 weeks at a daily dose of 0.6 mmol/kg (1.2 ml/kg), equivalent to the ordinary human dose of 0.1 mmol/kg, as recommended by the FDA [17]. Each group was divided into 2 subgroups based on the time-point for sample collection: 7 days (n = 6 per group) and 42 days (n = 7 per group) after the last injection. For the 7-day subgroup, 6 rats were euthanized for ICP-MS. For the 42-day subgroup, 6 rats underwent T1-weighted MRI and ICP-MS, and 1 rat was analyzed by transmission electron microscopy (TEM) (Figure 1).

### 2.3. MRI Protocol and Image Analysis

MRI acquisitions were performed under sodium pentobarbital anesthesia before the first GBCA injection and once a week until euthanasia (Figure 1). During the injection period, MRI was performed 3 days after 4 consecutive days of administration to ensure a 72-hour clearance period for Gd. MRI acquisitions were performed with an 8-channel wrist coil on a clinical 3.0-T Twin Speed Scanner (General Electric Medical Systems, Milwaukee, WI). T1-weighted MRI was performed using a fast spin echo sequence with the following parameters: repetition time (TR)/echo time (TE) = 600 ms/Min Full ms; field of view (FOV) = 6.0 cm; phase FOV = 1.00 cm; echo train length = 3; bandwidth = 31.25 kHz; matrix = 192×192; slice thickness = 1.0 mm; and number of excitations (NEX) = 2. Eleven slices were acquired to provide coverage from the olfactory bulb to the cerebellum.

All images were analyzed under blinded and randomized conditions. Quantitative evaluations of the DCN T1 signal intensity were performed by positioning the regions of interest (ROIs) over the bilateral DCN and cerebellum zones according to the anatomy of the rat brain [4, 18]. The signal intensity ratio was calculated as follows: DCN (the higher of the 2 sides)/cerebellum ratio [4].

### 2.4. Gd Concentration Analysis by ICP-MS

The animals were euthanized by an overdose of sodium pentobarbital anesthesia at the end of the clearance period. The heart was exposed, and cardiac perfusion with 0.9% saline solution was performed to remove excess blood from the brain. The whole brain was carefully isolated and was quickly dissected using the rat brain matrix (DSN-2; Institute of Materia Medica, China). The samples of the DCN, cerebellum, pons, olfactory bulb, frontal cortex, striatum, hippocampus, and thalamus were carefully extracted on ice according to the anatomical atlas of the rat brain [18].

The brain samples were weighed and sealed in a quartz tube immersed with 1.5 mL of concentrated nitric acid and were subjected to digestion in a microwave digester for 85 min (ETHOS One; Milestone, Bergamo, Italy). Each sample was then transferred to a polypropylene tube and diluted to 10 mL with ultra-purified water. Subsequently, the accumulation of the ^158^ Gd isotope in each sample was measured with ICP-MS (7700x; Agilent Technologies, Santa Clara, CA) (with an internal standard of indium 115). A standard curve of inorganic Gd (0.1-50 *μ*g/L) was used to monitor the response of the Gd concentration. The results were expressed in nanomoles (nmol) of Gd per gram of wet tissue weight.

### 2.5. Transmission Electron Microscopy

TEM was performed to characterize and localize the distribution of Gd deposits in both normal and diabetic rats. One rat of each 42-d subgroup was euthanized by an overdose of sodium pentobarbital. The brains were isolated and washed with ice-cold saline. The olfactory bulbs were isolated on ice as soon as possible, and 1-mm^3^ samples were collected and fixed with 2.5% glutaraldehyde at 4°C. Thereafter, the samples were rinsed in 0.1 mol/L Na-cacodylate buffer (pH=7.4) 3 times, fixed in 1% osmic acid for 2 h, and washed with saline. Subsequently, they were dehydrated through gradient ethanol and gradient acetone. The samples were then soaked in Epon812 epoxy resin overnight and underwent temperature drying and polymerization in an oven at 70°C for 24 h. Thereafter, ultrathin brain sections (0.1 *μ*m) were cut, stained with 2% lead citrate, and mounted on copper grids. The ultrastructures of olfactory bulbs were observed by TEM (HITACHI H-7650, Japan; acceleration voltage at 80 kV). The observed spots were analyzed by energy-dispersive X-ray spectroscopy (EDX) for elemental composition.

### 2.6. Statistical Analysis

The data are expressed as the means ± standard deviation (SD). Normality was assessed using the Shapiro-Wilk test. The Mann-Whitney U test was used to compare the blood glucose levels and cumulative injection dose of GBCA between normal and diabetic rats. For quantitative analyses of the T1 signal ratios, differences in groups at the same time-point were tested with 2-way analysis of variance (ANOVA) followed by the post hoc Bonferroni test for multiple comparisons. The effect of the diabetic status on Gd retention was analyzed with unpaired t test or the Mann-Whitney test. The Gd retentions of different parts of the brain were analyzed with 1-way ANOVA followed by Tukey's post hoc test or the Kruskal-Wallis test. The Gd concentrations of different GBCAs were analyzed with 1-way ANOVA, followed by Tukey's test or the Games-Howell test. The clearance of Gd from the brain was analyzed with unpaired t test or the Mann-Whitney test. Statistical analyses were performed using SPSS version 20.0 (IBM-SPSS, Inc., Chicago, IL, USA). A* p *value less than 0.05 was considered statistically significant.

## 3. Results

### 3.1. Animal Observations

All the rats survived the whole study. The blood glucose levels at the end of the research in all diabetic rats were greater than those in normal rats (24.6 ± 2.3 vs 5.2 ± 0.4 mmol/L, respectively;* p* < 0.001). No difference was found in the cumulative injection dose of GBCA (mmol) between the normal and diabetic groups (3.44 ± 0.26 vs 3.41 ± 0.31, respectively;* p* = 0.988).

### 3.2. Quantitative Analysis of T1-Signal Changes in the DCN

**Figure 2:**
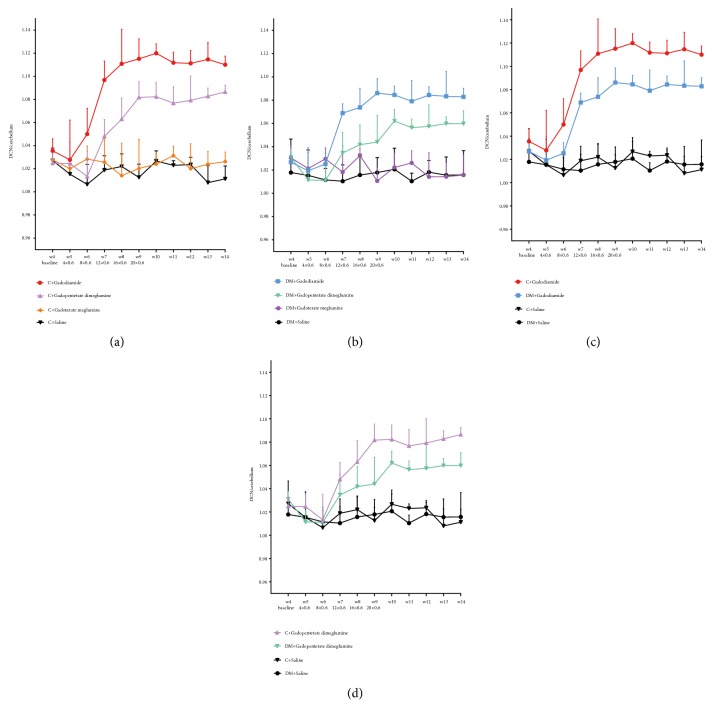
Quantitative analysis of the T1-weighted DCN to cerebellum signal ratio on weekly MR sequences. (a) Normal rats with different GBCAs. (b) Diabetic rats with different GBCAs. (c) Rats with linear gadodiamide. (d) Rats with linear gadopentetate dimeglumine. The error bars represent the SD of the DCN to cerebellum signal ratio. C = normal rats; DM = diabetic rats.

Normal and diabetic rats exhibited similar characteristics in the differences of the T1 signal intensity ratios among the 3 GBCA groups (Figures 2(a) and 2(b)). Significant T1 enhancement of DCN was observed in the gadodiamide group from the third week of the injection period (week 7) until the end of the study (week 14,* p *< 0.05) compared with that in the saline group and macrocyclic gadoterate meglumine group. Enhancement in the gadopentetate dimeglumine group was more progressive than that in the gadodiamide group during the injection period and treatment-free period. No such enhancement was observed in the macrocyclic gadoterate meglumine group compared with the saline group (*p *> 0.05).

Compared with those of normal rats, the T1 signal intensity ratios of the DCN/cerebellum in diabetic rats treated with gadodiamide were significantly lower during the injection period and treatment-free period (*p *< 0.001) (Figure 2(c)). Similarly, the signal ratios in diabetic rats treated with gadopentetate dimeglumine were relatively lower from the eighth week until the end of the study than those in normal rats (*p *< 0.001) (Figure 2(d)). In the gadoterate meglumine group, no significant difference was found in the T1 signal intensity ratio of the DCN/cerebellum in diabetic rats and normal rats, both at the baseline level (*p *> 0.05).

**Figure 3:**
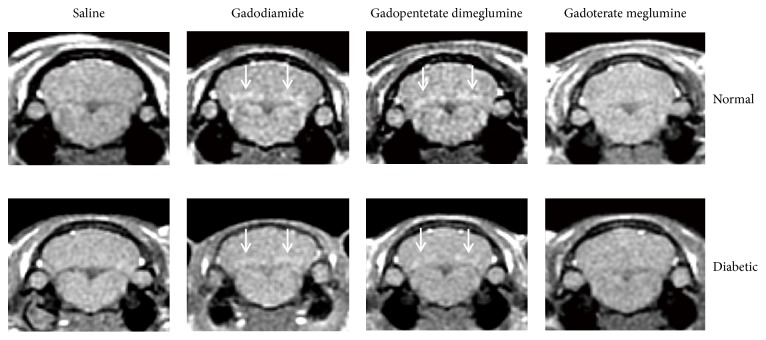
Representative T1-weighted MR images of the DCN in normal rats and diabetic rats 42 days after the last injections of saline, gadodiamide, gadopentetate dimeglumine, and gadoterate meglumine. Increased signal intensities of the DCN are shown with white arrows.

Representative T1-weighted MRI images of the normal and diabetic rats treated with saline, gadodiamide, gadopentetate dimeglumine, and gadoterate meglumine at the end of the treatment-free period are shown in Figure 3.

### 3.3. Gd Quantification in the Brain of Normal and Diabetic Rats by ICP-MS

**Figure 4:**
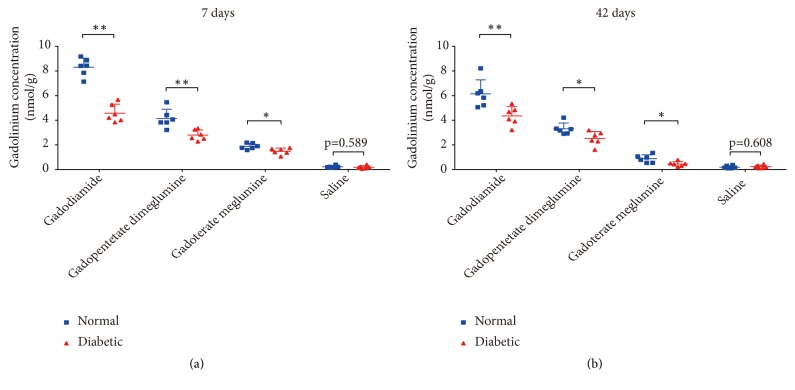
Average Gd concentrations determined by ICP-MS in the brain of normal and diabetic rats 7 days (a) and 42 days (b) after the last injections of gadodiamide, gadopentetate dimeglumine, gadoterate meglumine, and saline. All the data are expressed as the means ± SD (*∗ p *< 0.05; *∗∗ p *< 0.01).

In both time-point subgroups of the 2 linear groups and the 1 macrocyclic group, the average Gd retentions in the brain of diabetic rats were significantly lower than those in normal rats (Figure 4). The ratios of the average Gd concentration for normal versus diabetic rats in the 7-d subgroup and 42-d subgroup of gadodiamide were 1.8 and 1.4, respectively. For different brain areas, the highest ratios were up to 2 to 3 for the olfactory bulb and DCN.

**Figure 5:**
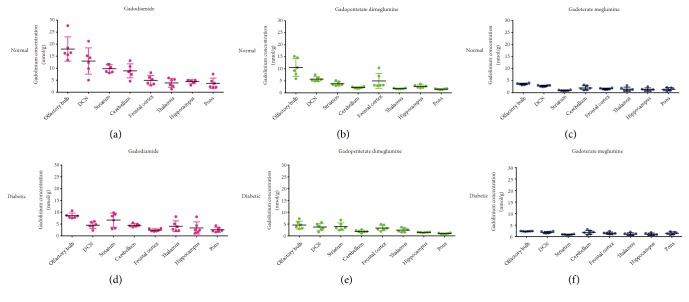
Gd concentrations in different brain areas of normal (a-c) and diabetic (d-f) rats 7 days after multiple injections of gadodiamide, gadopentetate dimeglumine, and gadoterate meglumine (mean ± SD).

**Figure 6:**
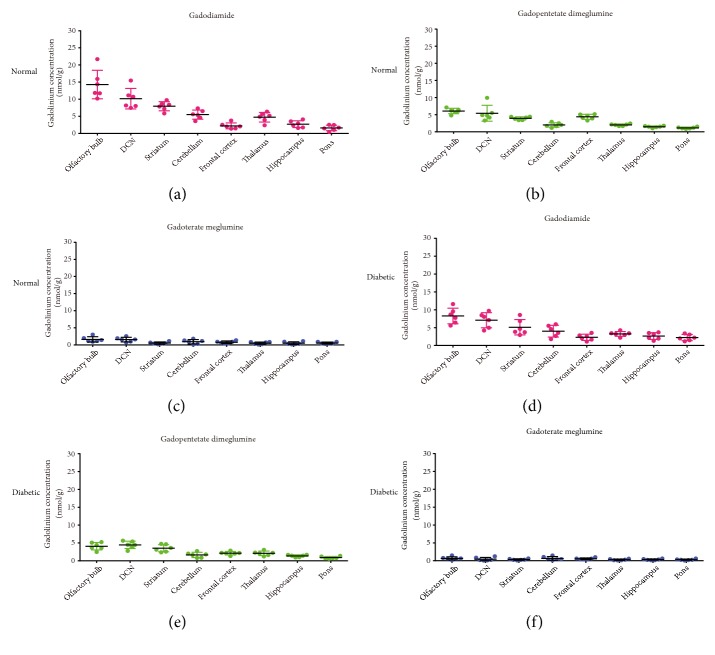
Gd concentrations in different brain areas of normal (a-c) and diabetic rats (d-f) 42 days after multiple injections of gadodiamide, gadopentetate dimeglumine, and gadoterate meglumine (mean ± SD).

The Gd concentrations in different brain areas at 7 d and 42 d are shown in Figures 5 and 6, respectively. In both the normal rats and diabetic rats, the Gd retentions varied among different brain tissues and exhibited similar distribution characteristics. The highest Gd retentions were in the olfactory bulb, DCN, and striatum with gadodiamide. For the macrocyclic gadoterate meglumine group, the Gd distributions were relatively more balanced. Compared with the 7-d subgroup, 42 days after dosing, the Gd distributions showed similar patterns among various brain areas.

**Figure 7:**
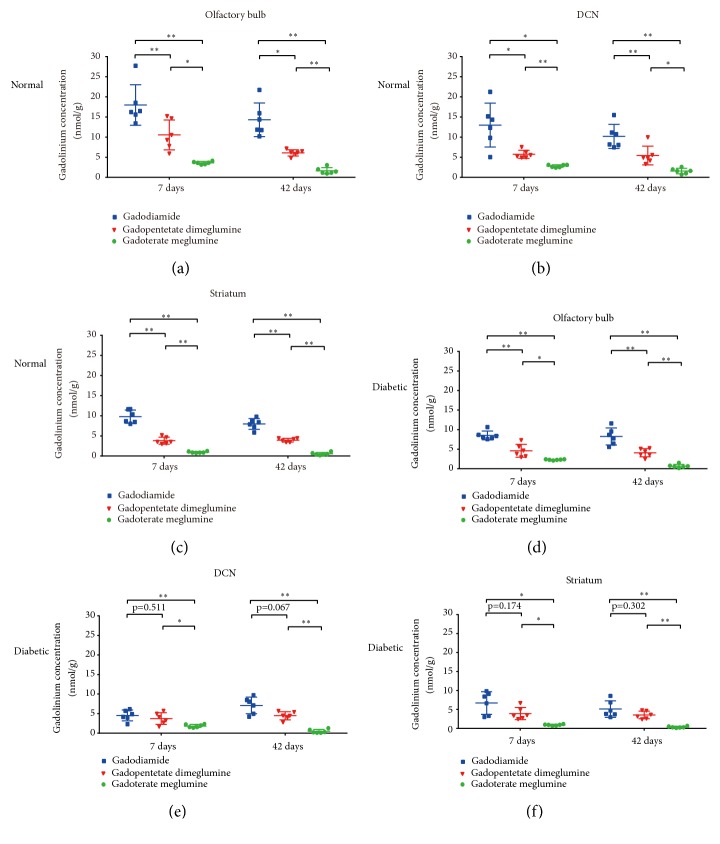
Gd concentrations in major brain areas with Gd retention in normal and diabetic rats after multiple injections of gadodiamide, gadopentetate dimeglumine, and gadoterate meglumine. (a-c) Gd concentrations in the olfactory bulb, DCN, and striatum of normal rats. (d-f) Gd concentrations in the olfactory bulb, DCN, and striatum of diabetic rats. All the data are expressed as the means ± SD (*∗ p *< 0.05; *∗∗ p *< 0.01).

In both the normal rats and diabetic rats, the highest average residual Gd retentions were measured in the gadodiamide group (8.31 ± 0.73 nmol/g tissue (7-d) and 6.15 ± 1.14 nmol/g tissue (42-d), for normal rats; 4.58 ± 0.73 nmol/g tissue (7-d) and 4.35 ± 0.76 nmol/g tissue (42-d), for diabetic rats), followed by the gadopentetate dimeglumine group (4.13 ± 0.76 nmol/g tissue (7-d) and 3.30 ± 0.48 nmol/g tissue (42-d) for normal rats; 2.79 ± 0.43 nmol/g tissue (7-d) and 2.52 ± 0.57 nmol/g tissue (42-d) for diabetic rats). By contrast, the Gd retentions with macrocyclic gadoterate meglumine were significantly lower (1.89 ± 0.23 nmol/g tissue (7-d) and 0.88 ± 0.32 nmol/g tissue (42-d), for normal rats; 1.48 ± 0.26 nmol/g tissue (7-d) and 0.45 ± 0.19 nmol/g tissue (42-d), for diabetic rats). Comparisons of the Gd concentrations with different GBCAs for major brain tissues associated with Gd retention are shown in Figure 7.

### 3.4. Clearance of Gd from the Brain in Normal Rats and Diabetic Rats

**Figure 8:**
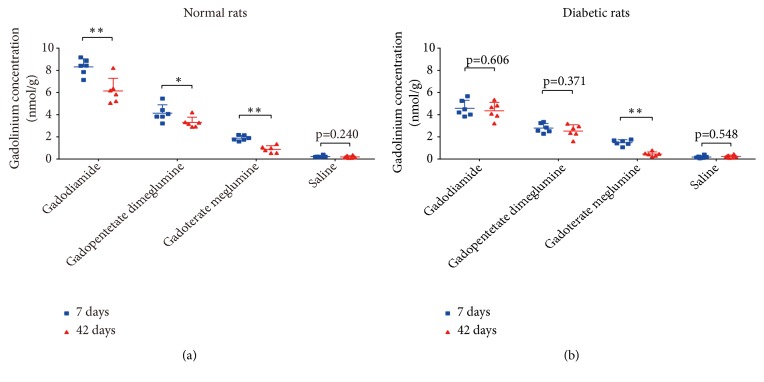
Clearance of Gd from the brain in normal (a) and diabetic rats (b) after multiple injections of gadodiamide, gadopentetate dimeglumine, gadoterate meglumine, and saline. All the data are expressed as the means ± SD (*∗ p *< 0.05; *∗∗ p *< 0.01).

In normal rats, compared with the residual Gd concentrations 7 days after the injection period, 42 days after injections, the average Gd concentrations were significantly decreased with both the linear GBCAs and macrocyclic gadoterate meglumine (8.31 ± 0.73 nmol/g tissue vs. 6.15 ± 1.14 nmol/g tissue,* p *< 0.01 for gadodiamide; 4.13 ± 0.76 nmol/g tissue vs. 3.30 ± 0.48 nmol/g tissue,* p *< 0.05 for gadopentetate dimeglumine; and 1.89 ± 0.23 nmol/g tissue vs. 0.88 ± 0.32 nmol/g tissue,* p *< 0.01 for gadoterate meglumine) (Figure 8(a)).

In diabetic rats, compared with the values 7 days after the injection period, the average Gd concentrations 42 days after injections with the 2 linear GBCAs showed no significant difference (4.58 ± 0.73 nmol/g tissue vs. 4.35 ± 0.76 nmol/g tissue,* p *= 0.606 for gadodiamide; 2.79 ± 0.43 nmol/g tissue vs. 2.52 ± 0.57 nmol/g tissue,* p *= 0.371 for gadopentetate dimeglumine). By contrast, for the macrocyclic gadoterate meglumine group, 42 days after injections, the average Gd concentration was significantly decreased (1.48 ± 0.26 nmol/g tissue vs. 0.45 ± 0.19 nmol/g tissue,* p *< 0.01) (Figure 8(b)).

### 3.5. Localization of Gd Deposits within the Brain

**Figure 9:**
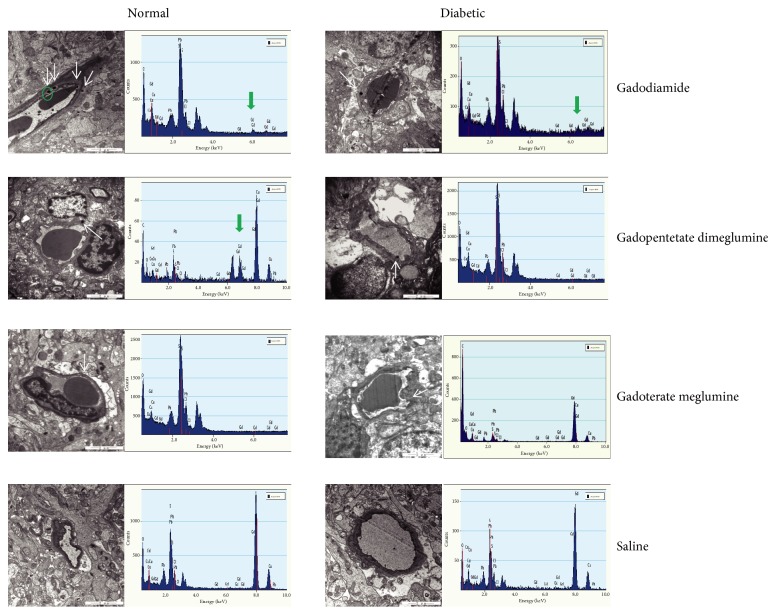
Localization of Gd deposits in the olfactory bulbs of normal and diabetic rats with TEM. Electron-dense granules are shown with white arrows. Obvious characteristic X-ray spectra of Gd are shown with green arrows.

During TEM evaluation, electron-dense granules were observed in the olfactory bulbs of both the linear gadodiamide group and linear gadopentetate dimeglumine group, including both the normal rats and diabetic rats, while no such electron-dense granules were found in both the normal and diabetic rats administered with macrocyclic gadoterate meglumine. Despite the observed electron-dense granule in diabetic rats treated with gadopentetate dimeglumine, the obvious characteristic X-ray spectra of Gd were not detected in EDX, indicating that the Gd concentration of the electron-dense granule was lower than the detection threshold of this qualitative analysis technique. The largest number of the Gd deposits was detected in normal rats treated with gadodiamide. These electron-dense Gd deposits were identified mainly within the endothelial wall of capillaries (Figure 9).

## 4. Discussion

This study revealed lower Gd retentions in the brain of diabetic rats than in normal rats, after repeated injections of two linear GBCAs—gadodiamide and gadopentetate dimeglumine—and one macrocyclic GBCA—gadoterate meglumine. DM is one of the most challenging endocrine disorders worldwide. As a common and basic disease, DM is often accompanied with other disorders, such as tumors, chronic liver diseases and vascular diseases requiring repeated enhanced MRI examinations. STZ-induced DM is considered one of the most potent chemically induced rat models of DM [19]. This model replicates the pathological changes and complications of diabetic patients and is widely used in studies on diabetes pathogenesis and therapeutics [16, 20].

Elimination of GBCAs from the body primarily depends on the renal function. Previous studies have shown that the half-life of GBCAs in healthy rats is approximately 18 min [21] and 1 to 2 h in healthy humans. DM has been demonstrated to lead to diabetic nephropathy in every third patient, which will impair the renal function [22]. DM is also associated with the impairment of the BBB [14] and glymphatic system, which has been shown to eliminate potential neurotoxic waste products, including GBCAs, from the interstitial space [15]. Therefore, we expected the brains of diabetic rats to be more vulnerable to Gd retention. However, in this study, the T1 signal intensity ratio of the DCN/cerebellum in diabetic rats was relatively lower than that in normal rats in both the linear GBCA groups, from the injection period until the end of the treatment-free period. Moreover, with the cumulative injection doses of GBCA (mmol) between the diabetic group and the control group being comparable, the average retained Gd concentration in the brain of diabetic rats in both time-point subgroups were significantly lower than that in normal rats, indicating that the residual Gd concentrations were decreased by the diabetic status. This finding is consistent with that in a previous study by Schleichert et al. [22] demonstrating that the prevalence of DM among patients with NSF was much lower than the expected United States Renal Data System rate of DM in all patients with end-stage renal disease (18.8% vs. 45%), indicating that DM patients may be less likely to develop Gd-induced NSF. Despite the impairment of the BBB and glymphatic system, DM may also influence Gd retention through other mechanisms.

To date, the mechanisms of Gd uptake and retention in the brain remain unclear. One hypothesis is transmetallation-induced dechelation and subsequent interaction with organic macromolecules [23]. Considering that the high T1-weighted signals are primarily in the dentate nucleus and thalamus where other metal ions, such as iron and calcium, also tend to show relatively higher concentrations, Gd uptake and accumulation may be mediated by biologic mechanisms, such as metal transporters [24]. In the diabetic status, the metabolism of glucose, lipid and protein is disordered [25–27], and the homeostasis of some metal transporter proteins is impacted [28]. The diabetic status may influence the expression of transporters concerned with Gd retention, which leads to lower Gd retention in diabetic rats than in normal rats. However, there is no confirmed evidence for this hypothesis, as speciation analysis has not yet been performed. Another possible speculation of Gd retention in the brain is the intact GBCA bound to organic macromolecules.

Two potential pathways of GBCA permeation and elimination in the brain are through the BBB [29] and glymphatic system [12]. Taoka et al. found that for animals administered GBCAs under anesthesia or during sleep, when the glymphatic system tends to be more active, Gd retention was lower [30]. This suggests that the glymphatic system may be involved in Gd retention in the brain. Interestingly, in the present study, for diabetic rats, the glymphatic system and BBB of which were considered to be impaired, the Gd concentrations in the brain were lower than those in normal rats. This indicates that the glymphatic system and BBB may not be the only two factors that influence Gd retention in the brain. The diabetic status may also influence the process of transporter mediation and subsequent released Gd or intact GBCA interaction with macromolecules, thus decreasing Gd retention in the brain. In the diabetic status, macromolecules such as proteins tend to be nonenzymatic glycosylated [31, 32]. The glycosylation of macromolecules in the diabetic brain may lead to a lower affinity for Gd. Intensive studies are needed to reveal the possible mechanism of the effect of the diabetic status on Gd retention in the brain.

The olfactory bulb had a much higher Gd concentration than most parts of the brain in both normal and diabetic rats. This finding is consistent with a previous study by Kartamihardja et al. [33] in which the Gd concentration was highest in the olfactory bulb in both the gadodiamide and gadoterate meglumine groups. The olfactory bulb has been proposed to be essential in the normal function of the glymphatic system. The glymphatic system is a recently described paravascular pathway for CSF and interstitial fluid exchange in the brain through which waste proteins and metabolites can be eliminated [34]. Recent studies have revealed that the glymphatic system is a potential pathway of GBCAs entering the brain [11]. After GBCAs were injected into the subarachnoid space of the cisterna magna in the mouse, GBCAs rapidly entered the brain parenchyma along the paravascular pathway from the basal artery to the olfactory artery and were transported especially into the olfactory bulb and cerebellum [35]. On the other hand, the CSF is drained from the subarachnoid space into the peripheral lymphatic system through the olfactory bulb and along the cranial and spinal nerves. The olfactory route is considered one of the most important efflux pathways for CSF in the brain [36]. This may explain the relatively higher concentrations of Gd in the olfactory bulb.

In addition to the olfactory bulb, the DCN and the striatum in both the healthy and diabetic rats also had relatively higher Gd concentrations in the brain in this study. Moreover, the signal intensity in the DCN on unenhanced T1-weighted MR images was increased in both the healthy rats and diabetic rats after multiple GBCA administrations and remained higher at the end of the 5 treatment-free weeks than in the saline group. These findings paralleled the increased T1 signal intensities in the dentate nucleus and globus pallidus in patients who were administered multiple doses of linear GBCAs [37]. Growing evidence has demonstrated that the dentate nucleus and globus pallidus are 2 targets for metals such as copper and zinc accumulation [38, 39]. Furthermore, it has been established that transmetallation with divalent metals in the dentate nucleus and globus pallidus occur with chronic exposure to manganese and lanthanum [40]. Lanthanum and Gd are both lanthanides in the periodic table and have similar properties. Therefore, Gd may also participate in transmetallation with divalent metals in the dentate nucleus and globus pallidus and deposit in these parts of the brain. For rats exposed to lanthanum, the activity of Ca(2+)-ATPase was inhibited, and the homeostasis of trace elements, enzymes and neurotransmitter systems in the brain was disturbed [40]. However, to date, there is no evidence that lanthanum ion has the same metabolism effects as dechelated Gd. Since no neurotoxicological consequence has been found in the patients and animals with Gd retention, dechelation probably occurs with the formation of a stable species, where Gd is secured and inactivated.

In the present study, T1 enhancement of DCN was observed exclusively in the 2 linear GBCA groups, with higher T1 signal intensity ratios in the gadodiamide group. On the other hand, in both normal and diabetic rats, the highest Gd retentions were measured in the gadodiamide group, followed by the gadopentetate dimeglumine group. However, the Gd retentions with macrocyclic gadoterate meglumine were significantly lower. Moreover, the TEM of Gd electron-dense foci along the endothelial wall in the olfactory bulb treated with linear GBCAs instead of macrocyclic gadoterate meglumine also confirmed the results of ICP-MS. These data are consistent with previous studies [4, 37] and indicate that the chemical structures of linear and macrocyclic GBCAs also influence Gd retention in the brain. Compared with the macrocyclic GBCAs, the linear structure may be more amenable to binding to macromolecules, leading to higher Gd retentions in the brain in the linear gadodiamide group and gadopentetate dimeglumine group.

This study revealed Gd retention in all analyzed parts of the brain in both the normal rats and diabetic rats 7 days after the 2 linear GBCA and 1 macrocyclic GBCA injections. At 7 d, the Gd concentrations in the brain were significantly lower for diabetic rats than for normal rats, indicating that in the short term, a much more clearable fraction of Gd was eliminated for diabetic rats. For normal rats, Gd was continuously partly eliminated from the brain in the healthy rats of both linear groups and macrocyclic gadoterate meglumine group at 42 d compared with that at 7 d. For diabetic rats, the average Gd concentrations in the 2 linear groups at 42 d were not declined compared with that at 7 d. It should be noted that although the decrease was smaller for diabetic rats compared to at 7 days in the 2 linear groups, the Gd concentrations in the brain at 42 d were also significantly lower for diabetic rats than for normal rats in both the linear and macrocyclic groups. These results indicated that the clearable fraction of Gd in the brain was eliminated faster in diabetic rats than in normal rats. Further investigations are needed to elucidate possible mechanisms of the diabetic status influencing the clearance of Gd retention in the brain.

The limitations of our study include the assessment of the effect of the diabetic status on Gd retention in the animal model only, and further work needs to be performed to investigate the impact of the diabetic status on Gd retention and clearance in clinical patients. In addition, the results of this study only reflect findings for the three GBCAs tested, and the retention and clearance may be different with other GBCAs, such as gadoteridol (ProHance), which has previously been shown to clear more rapidly from normal rat brain and body tissues [5, 41, 42], possibly because of unique molecular properties [41, 43]. Further molecular investigations are needed to reveal the possible mechanisms by which decreased Gd retention occurs in diabetic brain tissues.

## 5. Conclusions

In conclusion, compared with normal rats, the diabetic status decreased the residual Gd concentrations in the brain after multiple intravenous administrations of linear (gadodiamide and gadopentetate dimeglumine) and macrocyclic (gadoterate meglumine) GBCAs. The clearable fraction of Gd was eliminated faster in diabetic rats than in normal rats. Our study might provide insight into the safety issue of GBCA administration in diabetic patients and contribute to revealing the mechanism of Gd retention in the brain.

## Data Availability

The data used to support the findings of this study are available from the corresponding author upon request.
